# An assessment of the relationship between clinical utility and predictive ability measures and the impact of mean risk in the population

**DOI:** 10.1186/1471-2288-14-86

**Published:** 2014-07-03

**Authors:** Kevin McGeechan, Petra Macaskill, Les Irwig, Patrick MM Bossuyt

**Affiliations:** 1 Sydney School of Public Health, The University of Sydney, Sydney, Australia; 2 The Screening and Test Evaluation Program, The University of Sydney, Sydney, Australia; 3 Department of Clinical Epidemiology, Biostatistics and Bioinformatics, Academic Medical Centre (AMC), University of Amsterdam, Amsterdam, The Netherlands

**Keywords:** Biomarkers, Net reclassification improvement (NRI), Area under curve (AUC), Net benefit, Event free life years (EFLY), Risk assessment, Prediction

## Abstract

**Background:**

Measures of clinical utility (net benefit and event free life years) have been recommended in the assessment of a new predictor in a risk prediction model. However, it is not clear how they relate to the measures of predictive ability and reclassification, such as the c-statistic and Net Reclassification Improvement (NRI), or how these measures are affected by differences in mean risk between populations when a fixed cutpoint to define high risk is assumed.

**Methods:**

We examined the relationship between measures of clinical utility (net benefit, event free life years) and predictive ability (c-statistic, binary c-statistic, continuous NRI(0), NRI with two cutpoints, binary NRI) using simulated data and the Framingham dataset.

**Results:**

In the analysis of simulated data, the addition of a new predictor tended to result in more people being treated when the mean risk was less than the cutpoint, and fewer people being treated for mean risks beyond the cutpoint. The reclassification and clinical utility measures showed similar relationships with mean risk when the mean risk was less than the cutpoint and the baseline model was not strong. However, when the mean risk was greater than the cutpoint, or the baseline model was strong, the reclassification and clinical utility measures diverged in their relationship with mean risk.

Although the risk of CVD was lower for women compared to men in the Framingham dataset, the measures of predictive ability, reclassification and clinical utility were both larger for women. The difference in these results was, in part, due to the larger hazard ratio associated with the additional risk predictor (systolic blood pressure) for women.

**Conclusion:**

Measures such as the c-statistic and the measures of reclassification do not capture the consequences of implementing different prediction models. We do not recommend their use in evaluating which new predictors may be clinically useful in a particular population. We recommend that a measure such as net benefit or EFLY is calculated and, where appropriate, the measure is weighted to account for differences in the distribution of risks between the study population and the population in which the new predictors will be implemented.

## Background

Models that calculate the risk of disease are widely used to aid diagnosis and prognosis [1]. Examples of commonly used models include the Framingham Risk score for CVD and the Gail model for breast cancer [2,3]. However, the predictions provided by these models are not perfect and ways to improve the predictions are frequently proposed. One such method is to include additional predictors in the model [4]. Whether the additional predictors provide better predictions and how this is evaluated has been the subject of numerous articles in recent years [5-8].

If a new predictor is to be added to a prediction model then the benefits of including this new predictor must outweigh the costs; the new predictor must demonstrate clinical utility. Measures of clinical utility that have been proposed include the net benefit and event free life years (EFLYs) [6,9]. Several authors have suggested that such measures of clinical utility be calculated after the new predictor has demonstrated incremental predictive ability in terms of either an increase in the c-statistic, the continuous version of net reclassification improvement (NRI(>0)) or the categorical NRI [5,10-12]. This staged approach implies the predictive ability results provide an indication of the likely clinical utility results.

The c-statistic has been criticised as being insensitive to the effect of important new predictors [13]. Therefore it is questionable whether such an insensitive measure is of use in determining which new predictors should then be assessed in terms of clinical utility. The NRI(>0) has been proposed as a better measure of discrimination than the c-statistic when comparing predictors but how the NRI(>0) then relates to the clinical utility measures has not been examined [5]. If the measures of predictive ability do not correlate with the measures of clinical utility then it is doubtful whether they would be helpful in deciding which new predictors should be investigated further.

An additional concern is that these measures may behave differently as the mean risk of the population being studied changes. For example, the c-statistic is largely unaffected by the mean risk in the population whereas measures of reclassification may be affected by where the reclassification cutpoint is set in relation to the distribution of risk in the population [14]. The categorical NRI also implicitly weights the reclassification of cases and non-cases by the prevalence in the sample population [15]. The impact of changing cutpoints on net benefit has been examined recently [16], however, less attention has been paid to the situation where the cutpoint is fixed but the mean risk varies across the populations studied, a common situation when new cardiovascular risk predictors are assessed.

In the cardiovascular setting, the application of risk thresholds for treatment has been widely promoted for a number of years in guidelines across the world [17-20]. New predictors of cardiovascular disease have then been assessed using these fixed thresholds but in a wide variety of populations. For example, the Emerging Risk Factors Collaboration has brought together 104 prospective population-based studies across a number of countries, several of which are from North America [21]. The mean age of these North American cohort studies ranges from 54 to 78, and the percentage of males from 0% to 100% [6]. This indicates a range of mean risks, and differences in the distribution of risks, across studies in which the same threshold for treatment would be applied.

In this paper we examine how the measures of predictive ability (c-statistic, binary-statistic, NRI(0), NRI (with two cutpoints), binary NRI (at the upper cutpoint)) are related to the measures of clinical utility: net benefit and event free life years (EFLY) for assessing the effect of adding a new predictor to a model. We investigate how differences in the mean risk between populations affect these measures using simulated data and also using data from the Framingham Study where the mean risk of CVD differs for men and women [22].

## Methods

### Measures of predictive ability

For each of the measures we have chosen to calculate them at a common, fixed follow-up time of ten years consistent with the UK guidelines that apply CVD risk prediction models [17].

Sensitivity and specificity at ten years, assuming a fixed cutpoint were calculated using the following formulas.

$$\text{sensitivity}=\frac{\text{number}\;\text{true}\;\text{positives}}{\text{number}\;\text{with}\;\text{event}}$$

$$\mathrm{specificity}=\frac{\text{number}\;\text{true}\;\text{negatives}}{\text{number}\;\text{without}\;\text{event}}$$

where the numbers of true positives, true negatives and those with and without an event were estimated using the Kaplan-Meier estimates of the proportion surviving at ten years.

Harrell’s c-statistic is a measure of discrimination calculated using the formula –

$$c=\frac{\text{number}\;\text{of}\;\text{concordant}\;\text{pairs}+0.5\;\times \;\text{number}\;\text{of}\;\text{tied}\;\text{pairs}}{\text{total}\;\text{number}\;\text{of}\;\text{usable}\;\text{pairs}}$$

Each individual with an event before ten years is paired with every other person, irrespective of their event status. A pair is usable if their observed survival times differ, and the paired person had an event or their censoring time was greater than the survival time for the individual with the event. A usable pair is concordant if the predicted survival time is less for the member of the pair with the shorter observed survival time. A pair is tied if they have the same predicted survival time. People with events after ten years are considered censored at ten years [23].

The binary area under the ROC curve (or binary c-statistic) is a measure of discrimination at a particular cutpoint. It is calculated by averaging the sensitivity and specificity at that cutpoint when the new marker is added to the model. We calculated the difference in binary c-statistic using the formula

$$\begin{array}{c} \text{difference}\;\text{in}\;\text{binary}\;c-\text{statistic}=0.5\times (\text{change}\;\mathrm{in}\;\text{sensitivity}\;\\ \;\;\;+\;\text{change}\;\text{in}\;\text{specificity}) \end{array}$$

### Measures of reclassification

NRI(>0) and NRI(with two cutpoints) measure the amount of reclassification that occurs when the new predictor is added to a model [24]. The proportion of events and non-events correctly reclassified (reclassified up and down, respectively) are adjusted by the proportion of events and non-events incorrectly reclassified.

We calculated the NRI(>0) using the formula –

$$\begin{array}{l} \mathrm{NRI}\left(>0\right)=\frac{\left(P\left(\text{event}|\text{up}\right)\times n_{U}-P\left(\text{event}|\text{down}\right)\times n_{D}\right)}{\left(n\times P\left(\text{event}\right)\right)}\\ \;+\frac{\left(\left(1-P\left(\text{event}|\text{down}\right)\right)\times n_{D}-\left(1-P\left(\text{event}|\text{up}\right)\right)\times n_{U}\right)}{\left(n\;\times \left(1-P\left(\text{event}\right)\right)\right)} \end{array}$$

where *n* is the total number of people and the subscripts *U* and *D* indicate those reclassified up and down. The Kaplan-Meier estimates at ten years among all people, and those reclassified up or down, provide the probabilities (P).

We calculated the NRI(with two cutpoints) using the formula –

$$\begin{array}{c} \mathrm{NRI}\left(\text{with}\;\text{two}\;\text{cutpoints}\right)=\left({\hat{p}}_{\text{up},\;\text{events}}-\;{\hat{p}}_{\text{down},\;\text{events}}\right)\\ \;\;+\left({\hat{p}}_{\text{down},\;\text{non}-\text{events}}-\;{\hat{p}}_{\mathrm{up},\text{non}-\text{events}}\right) \end{array}$$

where $\hat{p}.,.$ is the proportion of events, and non-events, that are reclassified up, or down. As the data are censored the number of events and non-events are estimated from the Kaplan-Meier estimates at ten years for each of the cells in the reclassification table [25].

The binary NRI was also calculated. This has a single cutpoint which was set as the upper cutpoint from the NRI(with two cutpoints). The binary NRI is directly related to the binary c-statistic.

binaryNRI=2×differenceinbinaryc-statistic

### Measures of clinical utility

The net benefit provides a measure of the number of people correctly classified as having the outcome, adjusted for the number of people incorrectly classified as having the outcome, where the number of people incorrectly classified as having the outcome is weighted by the relative importance of a correct classification compared to an incorrect classification [26]. This weight is determined by the threshold probability at which people are classified as having the outcome. We calculated the Net Benefit using the formula –

$$\text{Net}\;\text{benefit}=\frac{\text{True}\;\text{positives}}{n}-\;\frac{\text{False}\;\text{positives}}{n}\left(\frac{p_{t}}{1-p_{t}}\right)$$

where the number of true positives and false positives are estimated using the Kaplan-Meier estimates of the percentage surviving at ten years among those with calculated risks greater than the threshold probability; *n* is the total number of people and *p*_*t*_ is the threshold that defines high risk.

The number of event free life years (EFLYs) was estimated using the methods described by Rapsomaniki and colleagues [6] and is based on the formula.

$$\text{EFLYs}\;\text{gained}\;\text{per}\;\text{person}\;\text{screened}=\;P\left(B\left(T\right)-C\left(T\right)\right)$$

where *P* is the proportion of those evaluated who are treated, *B(T)* is the benefit in terms of event free life years gained among those treated and *C*(*T*) is the costs for those treated, measured also relative to event free life years. Briefly, individuals with a calculated risk above a treatment threshold are assumed to have their risk reduced by treatment. This reduction in risk leads to a reduction in events and an increase in the total number of event free life years for the population within a given time period (here 10 years). Each gain in EFLY is assumed to have a monetary value. However, there are costs involved in treatment particularly for those who would not have experienced an event within the time period. Assuming a particular cost per EFLY, Rapsomaniki’s method deducts these costs in terms of EFLYs from the benefit obtained from the gain in EFLY of those treated.

As in Rapsomaniki’s paper, we set the reduction in risk due to treatment at 20% which was based on results from a meta-analysis [27]. The cost of treatment, in terms of EFLYs, is calculated assuming that the threshold for treatment is the optimal cutpoint in that benefits match costs at this point. Rapsomaniki and colleagues put a monetary value on this cost by relating it to the cost of one EFLY (£20,000) as proposed by the National Institute for Health and Clinical Excellence (NICE) [28].

Our main analysis focused on the upper cutpoint of 20% risk at ten years which is used in the UK CVD prevention guidelines [17]. We also repeated our analyses using upper cutpoints of 10% and 50%. The lower cutpoint in the calculation of the NRI categorical for these analyses were arbitrarily set at 5% and 25%, respectively.

### Simulated data

For our simulations we generated survival times that followed a Cox-exponential survival model using the formula [29].

$$T=\frac{-\log\;\left(U\right)}{\lambda \;\exp\;\left(\beta_{1}x_{1}+\beta_{2}x_{2}\right)}$$

Where *U* is a uniform random number between 0 and 1, *λ* is the baseline hazard rate which was varied to produce datasets with mean risks at ten years distributed between 0% and 100%. The variables *x*_*1*_ and *x*_*2*_ each had standard Normal distributions and were independent of each other. We carried out separate series of simulations by varying the coefficient *β*_*1*_ from a hazard ratio of 1.5 per 1 standard deviation increase (weak baseline model) to 3 (medium baseline model) to 6 (strong baseline model) and by varying coefficient *β*_*2*_ of the second covariate to produce hazard ratios of 1.2 (weak predictor), 2 (medium predictor) and 3 (strong predictor). Note the hazard ratios derived from the Framingham dataset for the traditional CVD risk factors of age, SBP and total cholesterol ranged from 1.25 to 2.04, per one standard deviation increase (Additional file 1: Table S1). Since we have assumed a constant hazard ratio across simulated datasets that have different mean risks, the odds ratio calculated for events occurring before ten years will not be constant across the datasets [30]. The estimated odds ratio is similar to the hazard ratio if the mean risk is small, but the odds ratio increasingly overestimates the hazard ratio as the mean risk increases.

We also generated censoring times that followed an exponential distribution with a 10% risk of being censored at ten years. If the censoring time was less than the survival time the observation was considered censored at the censoring time. The proportion censored decreased from 10% to approximately 2.5% as the mean risk increased. Each simulation dataset contained 10,000 observations. We simulated 1,000 datasets for each combination of baseline model and additional predictor.

For each of the simulated datasets the measures of predictive ability and clinical utility were calculated comparing models without and with the second variable. We then plotted the proportion of people classified as high risk (above the upper cutpoint) by each of the two models classified by the mean calculated risk of an event at ten years for that dataset. The mean risk was calculated from the model containing both covariates. We plotted the measures of predictive ability and clinical utility against the mean risk and applied a cubic spline smoother.

### Empirical data

We obtained data from the Framingham Heart Study on the people included in the analysis that resulted in the 2008 Framingham risk equation [22]. At the initial visit, blood pressure, serum total cholesterol, HDL, smoking status, diabetes status and use of anti-hypertensive medication were recorded using standard methods. All study participants were free of prevalent CVD at the initial visit and were under continuous surveillance for the development of cardiovascular events and death. Maximum follow-up was 12 years.

We fitted two Cox proportional hazards models to the Framingham dataset consisting of the variables that were included in the proposed general CVD risk prediction model [22]. The first model included age, total cholesterol, high density lipoprotein, smoking status, diabetes status and use of antihypertensive medication. The second model included all of these variables, plus systolic blood pressure (SBP). We carried out separate analyses for men and women as the risk of CVD differs between men and women.

We compared the models without SBP and with SBP using the following measures: change in c-statistic, binary c-statistic, NRI(>0), NRI(10%, 20%), binary NRI (20%), net benefit and the event free life years (EFLY). Ninety-five percent confidence intervals were calculated for these measures using 2000 bootstrap samples. We used a treatment cutpoint of 20% for the calculations of the measures net benefit and EFLYs (and 10%, 20% for the NRI(10%, 20%)) to match current cardiovascular disease (CVD) prevention guidelines [17]. We assumed that for treated people their risk of CVD would be reduced by 20% based on the meta-analysis reported in the paper by Rapsomaniki that introduced the EFLY [6].

## Results

### Simulated data

**Figure 1:**
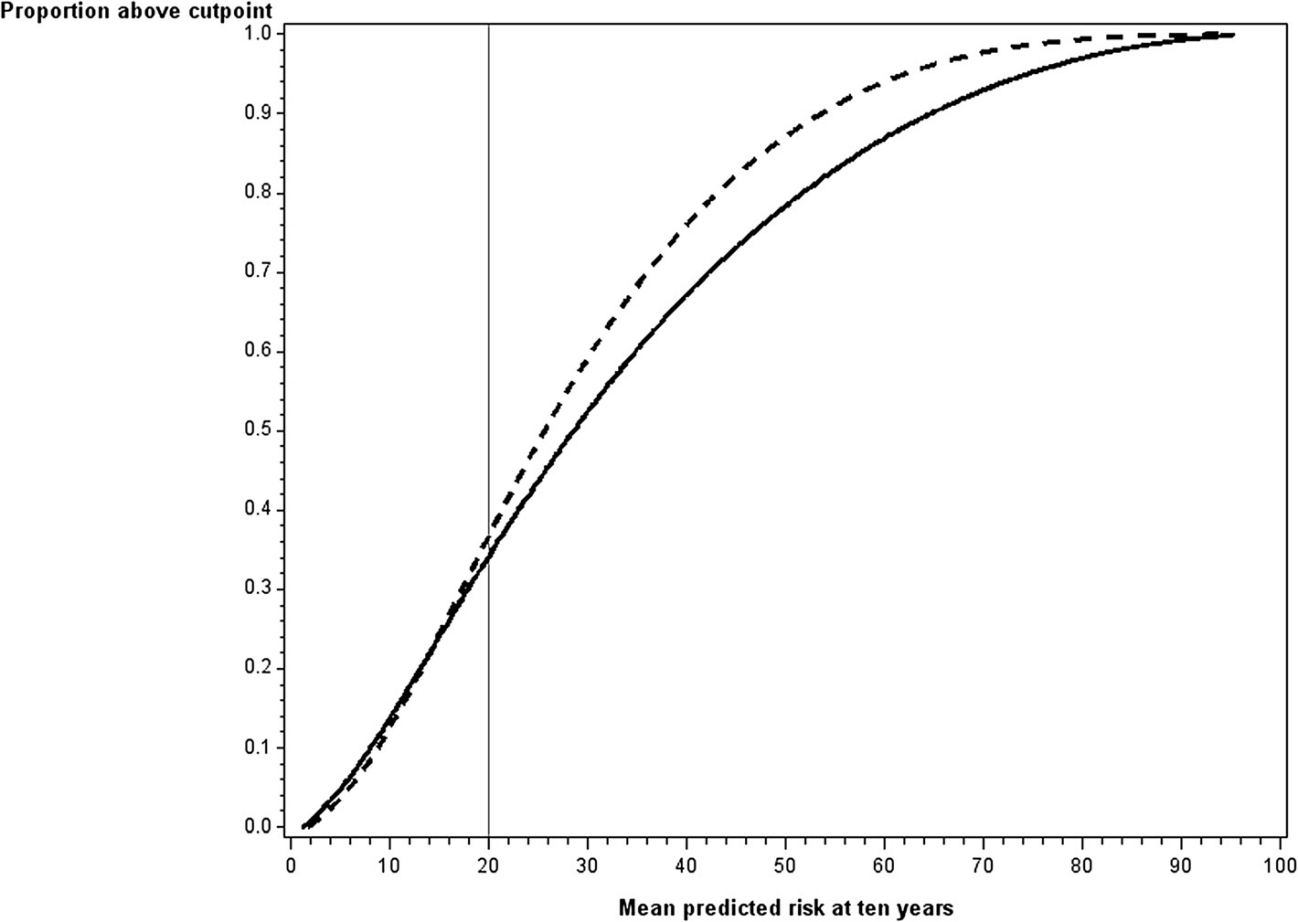
**Proportion of people above cutpoint assuming predictions based on the Base model or the Base Model + New Predictor, assuming a hazard ratio of 2 for the New Predictor.** Broken line = Proportion above cutpoint for baseline model. Solid line = Proportion above cutpoint for baseline model plus new predictor.

**Figure 2:**
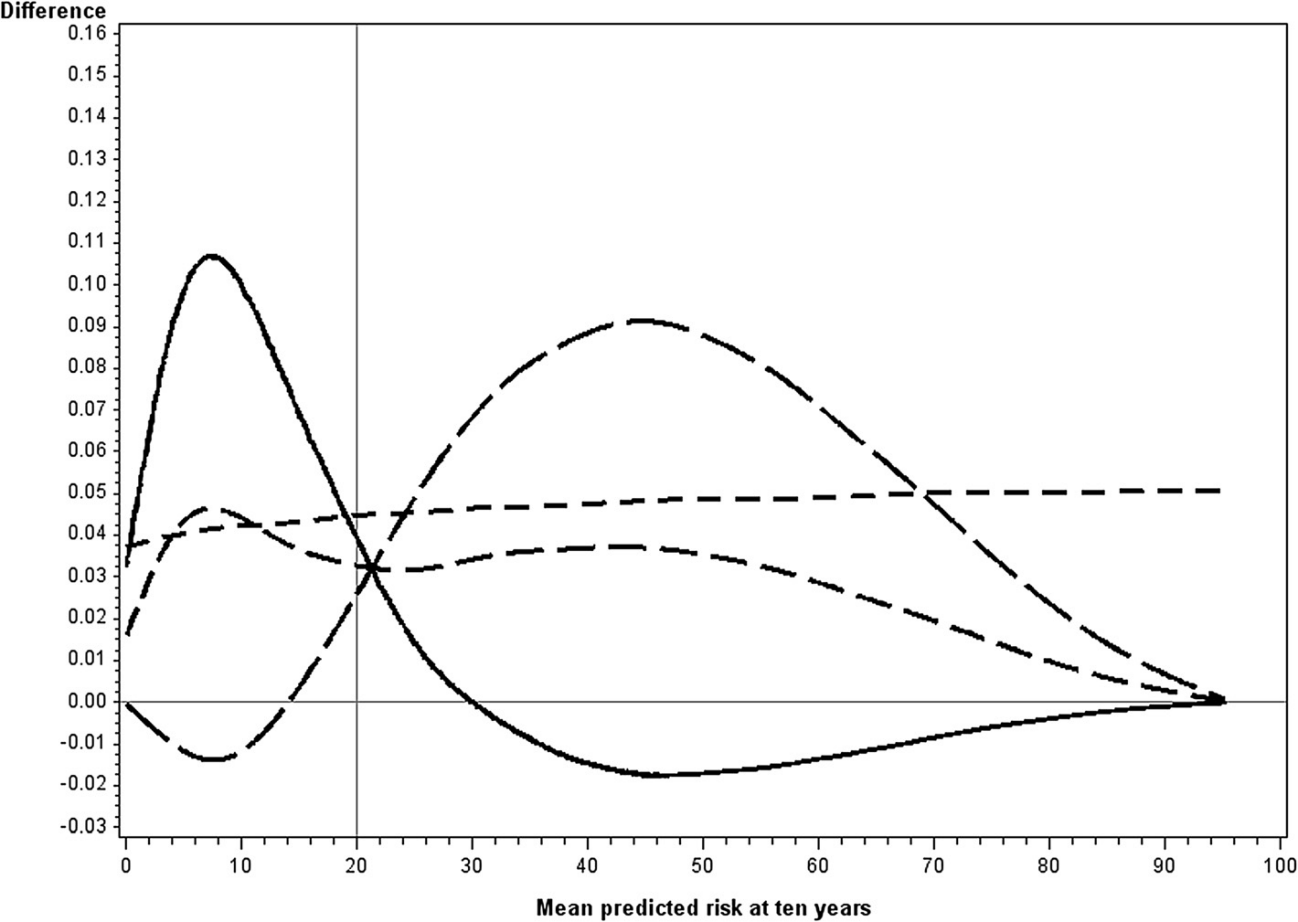
**Differences in measures of predictive ability when a new predictor (hazard ratio = 2) is added to the baseline model (medium).** Legend:  Sensitivity,  Specificity.  c-statistic  binary c-statistic.

The simulation results presented focus on the scenario where a new predictor with a hazard ratio of 2 per 1 standard deviation (medium new predictor) was added to a baseline model whose covariate had a hazard ratio of 3 per 1 standard deviation (medium baseline model) and the cutpoint of 20% defined high risk. The results obtained for other combinations of new predictor, baseline model and cutpoint were similar for the majority of combinations. We highlight below where important differences arose.In Figure 1 the difference in proportion classified as high risk (risk greater than 20% at ten years) is shown when a new predictor with a hazard ratio of 2 per 1 standard deviation (medium new predictor) was added to a baseline model whose covariate had a hazard ratio of 3 per 1 standard deviation (medium baseline model). There was a slightly higher proportion of people classified as high risk when the model with the new predictor was applied than when the baseline model was applied for the majority of datasets whose mean risk was below the cutpoint. For datasets where the mean risk was near or above the cutpoint, a higher proportion were classified as high risk by the baseline model. Similar results were obtained when the hazard ratio for the new predictor and the baseline model were varied. When the new predictor was weak (hazard ratio of 1.2) there was very little difference in the proportions classified as high risk across all levels of mean risk.Figure 2 shows that the model with the new predictor had consistently larger c-statistics than the base model. The increase in the c-statistic ranged from 0.04 to 0.05 when the new predictor was moderate (hazard ratio of 2). As the mean risk increased there was a small increase in the difference in the c-statistic. Although in the simulated datasets the hazard ratio was constant as the mean risk increased, the odds ratio increased. At a mean risk of 5% the average odds ratio in the simulated datasets was 2.08, at 10% 2.21, at 25% 2.34, at 50% 2.61 and at 90% 3.14.Differences in sensitivity increased and then decreased as the mean risk increased from zero to the cutpoint (Figure 2). The maximum improvement in sensitivity with the addition of the new predictor occurred when the mean risk was approximately half of the cutpoint. At this point, and for the majority of the range of mean risks below the cutpoint, specificity was lower with the addition of the new predictor. The model with the new predictor had lower sensitivity when the mean risk was above approximately 1.5 to 2 times the cutpoint and as the strength of the baseline model increased, the point at which the model with the new predictor had lower sensitivity increased. In contrast the difference in specificity increased with mean risk achieving a maximum at approximately the point when mean risk was double the cutpoint for a baseline model of medium strength. The point at which the maximum difference in specificity was achieved increased with the strength of the baseline model, at which point the decrease in sensitivity with the addition of the new predictor was at its greatest.

The difference in c-binary (which is the average of the differences in sensitivity and specificity) peaked at two points before approaching zero as the mean risk increased. The first peak corresponded to the maximum difference in sensitivity which happened at approximately half of the cutpoint, and the second corresponded to the maximum difference in specificity which happened above the cutpoint.

**Figure 3:**
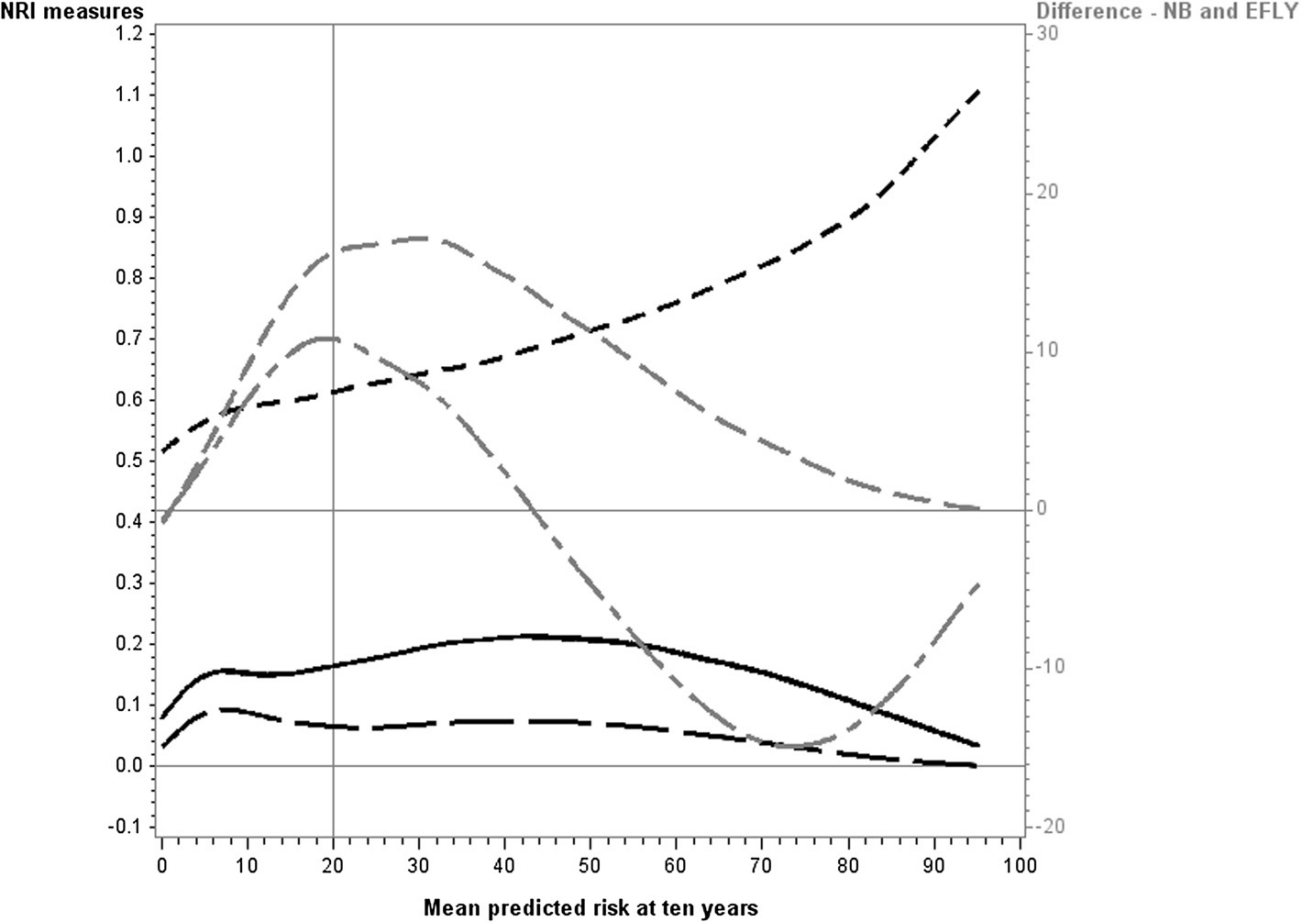
**Reclassification measures and differences in Net Benefit and Event Free Life Years when new predictor (hazard ratio =2) is added to baseline model (medium strength).** Legend:  NRI two cutpoints,  binary NRI  continuous NRI  Net Benefit  Event Free Life Years (EFLY). Note: This graph has two vertical axes. The NRI measures are plotted against the left vertical axis and the EFLY and Net Benefit are plotted against the right vertical axis.

Similar patterns were observed for all combinations of baseline model and new predictor for each of the measures: differences in c-statistic, sensitivity, specificity and binary c-statistic.The pattern observed for the difference in c-binary was reflected in the pattern for NRI binary, as these measures have a direct relationship (Figure 3). The NRI(10%, 20%) showed a similar pattern in that it reached two peaks, however the second peak above the cutpoint was larger than the peak below the cutpoint. As with NRI binary the NRI(10%, 20%) approached zero as the mean risk increased. The NRI-continuous, in contrast, continued to increase with increasing mean risk. As with the increase observed in the c-statistic, this is due to underlying odds ratio increasing with increasing mean risk when the hazard ratio remains constant.

**Figure 4:**
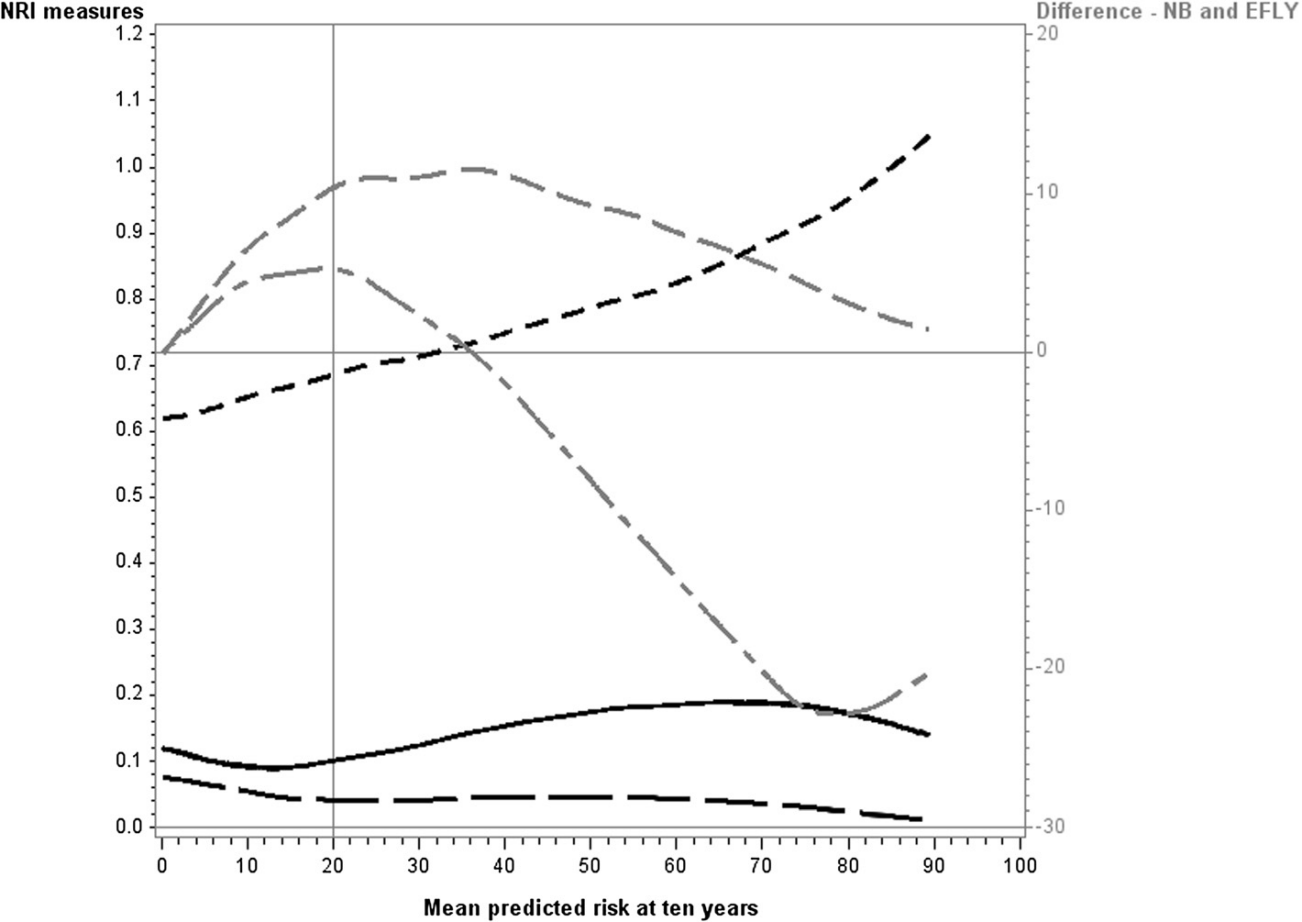
**Reclassification measures and differences in Net Benefit and Event Free Life Years when new predictor (hazard ratio =2) is added to baseline model (strong).** Legend:  NRI two cutpoints,  binary NRI  continuous NRI  Net Benefit  Event Free Life Years (EFLY). Note: This graph has two vertical axes. The NRI measures are plotted against the left vertical axis and the EFLY and Net Benefit are plotted against the right vertical axis.

**Figure 5:**
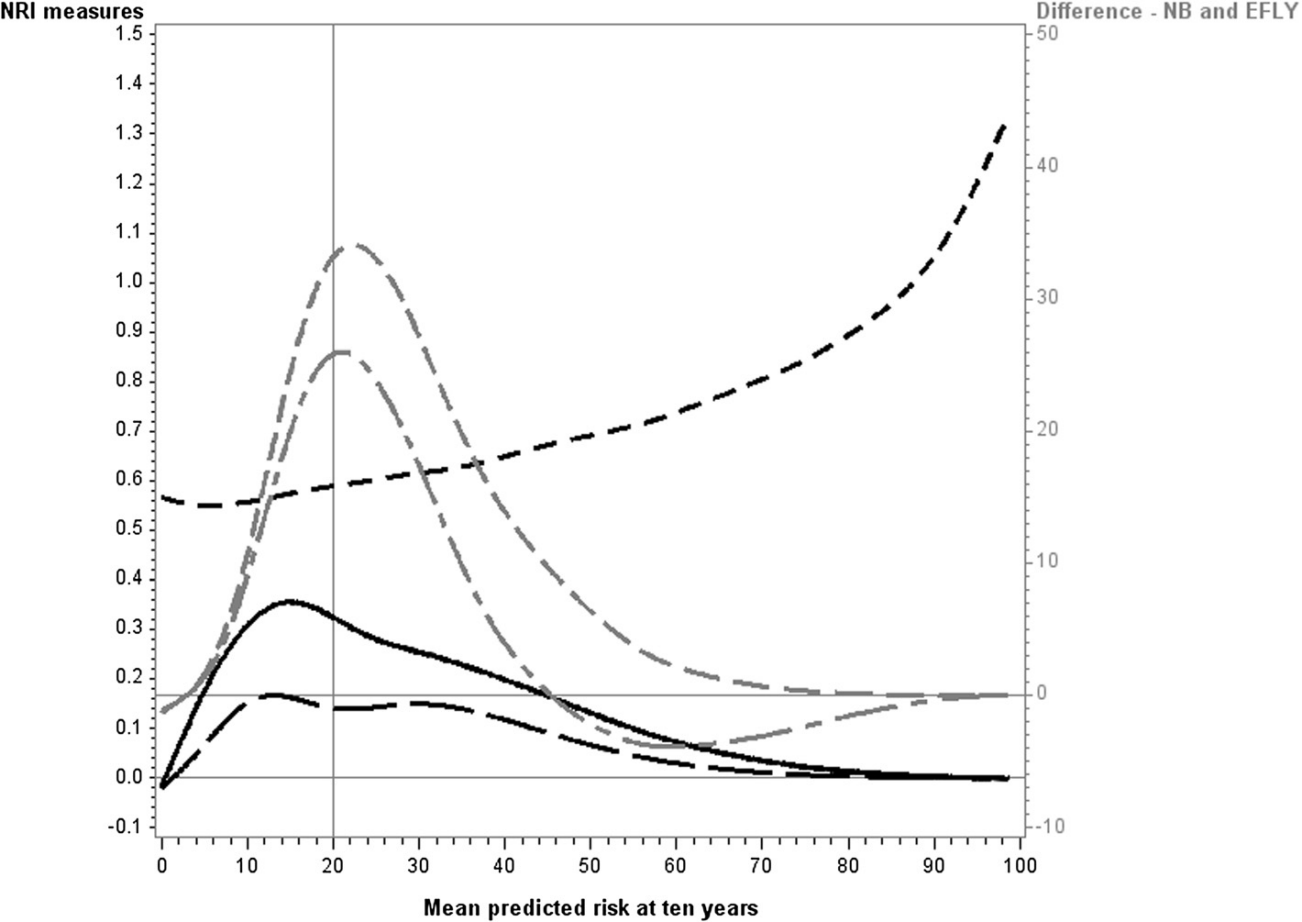
**Reclassification measures and differences in Net Benefit and Event Free Life Years when new predictor (hazard ratio =2) is added to baseline model (weak).** Legend:  NRI two cutpoints,  binary NRI  continuous NRI.  Net Benefit  Event Free Life Years (EFLY). Note: This graph has two vertical axes. The NRI measures are plotted against the left vertical axis and the EFLY and Net Benefit are plotted against the right vertical axis.

The two measures of clinical utility (difference in Net benefit and difference in EFLY) showed similar patterns in their relationships with mean risk. They initially increased from zero as the mean risk approached the cutpoint and attained a maximum at approximately the cutpoint. When mean risk was higher than the cutpoint, the two measures dropped with the difference in Net Benefit approaching zero with increasing risk. The difference in EFLY dropped below zero above the cutpoint but then approached zero as the mean risk increased.There was some variation in these patterns depending on the strength of the baseline model. When the baseline model was strong (Figure 4) similar results were obtained for the differences in Net Benefit and EFLY, however for the NRI measures they initially decreased for mean risks below the cutpoint. When the baseline model was weak (Figure 5), the second peak for the NRI categorical and NRI binary was less pronounced and tended to be lower than that for the first peak.

**Figure 6:**
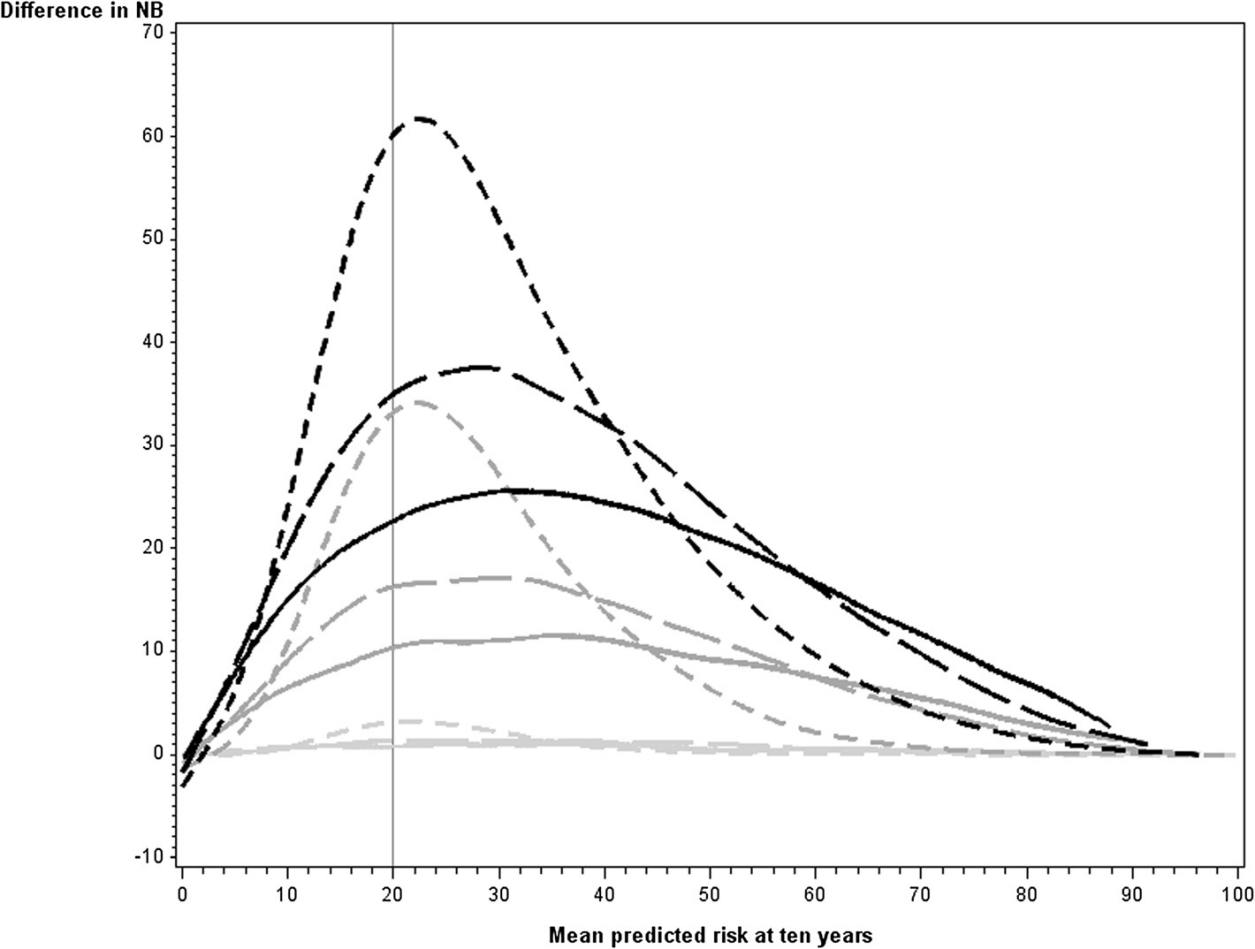
**Difference in Net Benefit for selected combinations of baseline model and new predictor.** Legend:  Strong baseline + strong new predictor  Medium baseline + strong new predictor.  Weak baseline + strong new predictor.  Strong baseline + medium new predictor  Medium baseline + medium new predictor.  Weak baseline + medium new predictor.  Strong baseline + weak new predictor  Medium baseline + weak new predictor.  Weak baseline + weak new predictor. Abbreviations: NRI = Net Reclassification Improvement, NB = Net Benefit, EFLY = Event Free Life Years.

We found the same general relationships between the different measures and the mean risk when the alternative cutpoints defining high risk of 10% and 50% were applied.Figure 6 displays the difference in Net Benefit plotted against the mean risk for various combinations of baseline model and new predictor. When the new predictor was weak the difference in net benefit was minimal at all levels of mean risk no matter the strength of the baseline model. The difference in net benefit was higher when a new predictor was added to a weaker model than when it was added to a stronger model. This held for both medium and strong new predictors. However, a medium new predictor when added to a weak model produced a higher difference in net benefit near the cutpoint than a strong new predictor when added to a strong model.

### Empirical data

There were 3969 men and 4522 women included in the Framingham dataset. The mean calculated risk of CVD at 10 years was 15.6% for men and 8.2% for women.

As expected, there was very strong evidence that systolic blood pressure was related to the risk of CVD after adjusting for the other risk factors (Table 1). The hazard ratio (per 20mmHg increase) for systolic blood pressure was greater for women (1.48) compared to men (1.30).

**Table 1:** Hazard ratios for the addition of systolic blood pressure to models predicting CVD for men and women in Framingham study

	**Men**		**Women**	
	**Base model**	**Base model + systolic blood pressure**	**Base model**	**Base model + systolic blood pressure**
	**Hazard ratio (95% CI)**	**Hazard ratio (95% CI)**	**Hazard ratio (95% CI)**	**Hazard ratio (95% CI)**
Age (per 10 year increase)	1.94 (1.80, 2.01)	1.80 (1.67, 1.95)	1.83 (1.66, 2.03)	1.56 (1.40, 1.74)
Total cholesterol (per 40 increase)	1.25 (1.17, 1.35)	1.23 (1.15, 1.32)	1.26 (1.16, 1.37)	1.23 (1.13, 1.33)
HDL (per 10 increase)	0.83 (0.77, 0.88)	0.82 (0.77, 0.87)	0.88 (0.82, 0.94)	0.88 (0.83, 0.94)
Hypertensive medication	1.72 (1.41, 2.08)	1.45 (1.18, 1.77)	1.76 (1.41, 2.19)	1.31 (1.04, 1.65)
Current smoker	1.91 (1.64, 2.22)	1.93 (1.66, 2.24)	1.71 (1.40, 2.07)	1.72 (1.42, 2.09)
Diabetes	1.89 (1.53, 2.34)	1.77 (1.42, 2.19)	2.14 (1.60, 2.86)	2.07 (1.55, 2.77)
Systolic blood pressure (per 20mmHg increase)		1.30 (1.20, 1.41)		1.48 (1.34, 1.62)

Among men, both sensitivity and specificity increased when systolic blood pressure was added to the model, whereas for women there was an increase in sensitivity but a decrease in specificity. This is consistent with the results from the simulations where datasets whose mean risk was approximately half the cutpoint showed a decrease in specificity and an increase in sensitivity. There was an increase in all the measures (except the NRI for women) to assess a new predictor for both men and women (Table 2) with the increases being greater for women. Although women had a mean risk lower than men this was compensated for by the greater hazard ratio for systolic blood pressure among women.

**Table 2:** Change in measures with addition of systolic blood pressure to models predicting cardiovascular disease for men and women

	**Men**			**Women**		
	**Base Model**	**Base Model + SBP**	**Difference**	**Base Model**	**Base Model + SBP**	**Difference (95% CI)**
Sensitivity	0.575	0.585	0.010 (-0.17, 0.033)	0.280	0.344	0.064 (0.006, 0.099)
Specificity	0.729	0.734	0.005 (-0.003, 0.016)	0.921	0.909	-0.013 (-0.020, -0.003)
c-binary	0.652	0.655	0.008 (-005, 0.018)	0.601	0.626	0.025 (0.000, 0.041)
c-statistic	0.751	0.758	0.007 (0.003, 0.012)	0.766	0.782	0.016 (0.009, 0.024)
NRI binary*			0.015 (-0.009, 0.036)			0.051 (0.000, 0.082)
NRI continuous*			0.170 (0.071, 0.267)			0.306 (0.176, 0.419)
NRI categorical*			0.028 (-0.009, 0.063)			0.091(0.010, 0.129)
Net benefit difference (per 1000 evaluated)	44.0	47.5	3.5 (-1.6, 7.7)	8.7	12.1	3.3 (-1.3, 6.4)
Event Free Life Years difference (per 1000 evaluated)	32.1	34.1	2.0 (-1.8, 5.4)	3.8	7.5	3.6 (-0.4, 6.3)

*Abbreviation*: *SBP* systolic blood pressure.

*There are no separate NRI measures for the baseline and the expanded model. They are calculated as an overall measure comparing two models.

## Discussion

We have described how the measures of predictive ability, reclassification and clinical utility used to assess a new predictor in a model depend upon the mean risk of the population. We have also demonstrated that the reclassification measures exhibit a different relationship with the mean risk than the clinical utility measures. The continuous NRI increases with increasing mean risk; the NRI categorical with two cutpoints often peaks at two points; whereas the net Benefit and EFLY peak once close to the cutpoint and then generally decrease to zero as the mean risk increases.

In the Framingham Study the mean risk of CVD was higher for men than for women, and also closer to the upper cutpoint of 20%. Based on this, we may have expected the measures of predictive ability, reclassification and clinical utility to be higher among men. However the hazard ratio for systolic blood pressure when it was added to the model was higher for women compared to men, and this compensated for the lower mean risk among women. In a recent review of several new predictors of cardiovascular disease, Paynter and colleagues have also highlighted that results may differ between men and women due to differences in effect sizes of new predictors as well as the strength of the baseline model and the mean risk in the study sample [31].

In our simulations we observed that as the mean risk increased the NRI(>0), and the change in the c-statistic, also increased. In the paper that introduced the NRI(>0) Pencina suggested that one of the benefits of this measure was that it was not affected by the event rates in the population [24]. Our simulations, where we assumed a constant hazard rate, indicate that the NRI(>0) increases as the event rate (the mean risk) increases for event rates above the cutpoint. The NRI(>0), as with the change in the c-statistic, is unaffected by event rates only if the odds ratio does not vary. However, as we have demonstrated, if the hazard ratio is assumed to be the same in populations with different event rates (a common assumption in cohort studies of cardiovascular outcomes) then the NRI(>0) will increase with increasing event rate.

In our simulations, when the mean risk in the population was less than the cutpoint the measures of reclassification and clinical utility were generally consistent with each other and increased as the mean risk increased. However, beyond this cutpoint the measures diverged. The reclassification measures continued to increase while the clinical utility measures decreased, although the NRI binary and NRI(with two cutpoints) did eventually decrease. Similar patterns were also observed by Van Calster and others when they varied the cutpoint and assumed a fixed mean risk; as the cutpoint moved away from mean risk the reclassification measures provided a more optimistic view of the new predictor compared to that provided by the difference in net benefit [16].

The clinical utility measures, difference in EFLY and difference in Net Benefit, achieved a maximum value at approximately the point where the threshold for treatment equaled the mean risk in the population, as expected [32]. However, we observed a divergence in the clinical utility measures in our simulations as the mean risk increased. This is attributable to differences between the two measures in terms of how benefits and costs are counted and the weights given to benefits and costs in populations with different mean risks.

When a new predictor is added to a model, the difference in EFLY is measured in terms of event free life years. An event free life year gained has the same value whether it occurs in a high risk or low risk population. In contrast, the difference in Net Benefit is measured in units of true positives, adjusted for false positives, with the weighting of false positives relative to true positives determined by the cutpoint defining high risk. However, the actual value of a true positive will differ in populations with different mean risks since the number of event free life years gained will be greater for an individual from a high risk population compared to a low risk population. Also, a false positive will have a greater cost in a low risk population than a high risk population as the survival time, and hence, treatment time, will be greater.

Although there are issues in using the Net Benefit when accounting for costs and benefits over a specific time period, there are also issues in the calculation of costs and benefits for the EFLY. Possible heterogeneity in treatment effects across patient subgroups is not accounted for in the EFLY. Also, the calculation of the EFLY assumes that the chosen cutpoint is the ‘optimal’ cutpoint in that costs equal benefits at this point; the cost of treatment, in terms of event free life years, is then calculated based on this assumption. Rapsomaniki and colleagues acknowledge that many factors, other than the costs and benefits they account for in their EFLY calculations, are considered when a particular cutpoint is chosen [6]. However, their assumption avoids the problem of an irrational choice of cutpoint resulting in a poorer model being favoured [6].

In previous papers the relationship between choice of cutpoint and the measures of reclassification and the difference in Net Benefit has been described when the mean risk in the population is fixed [14,16,33]. We observed similar results when the mean risk in the population varies but the cutpoint is fixed. The scenario we have described is the one more commonly encountered in the evaluation of new predictors of cardiovascular events. For example, the Emerging Risk factor Collaboration (EFRC) brings together several cohort studies from the same country which have different mean risks but where the same guidelines and cutpoints for defining high risk would apply. As each of the measures we have examined are in some way affected by the mean risk in the study population this must be taken into account when comparisons are made between different studies whose mean risk varies, or when the mean risk in the study population differs from the population in which a new predictor will ultimately be implemented.

A number of methods have been proposed to allow for these differences. Where the study data arise from a matched case control study Pepe has proposed a method for calculating an adjusted c-statistic that takes into account the greater similarity in risk between cases and controls that arises from matching [34]. The ERFC applied age-sex specific measures of reclassification observed in their study population to the standard European population to estimate the amount of reclassification that would occur in this standard population [35,36]. However, this relies upon having a large enough study population to provide reliable estimates of reclassification in each age-sex stratum. If the data arise from a case control study, Rousson suggests reweighting the proportions of cases and controls to match the proportions found in the parent population [37].

## Conclusion

There have been a number of recent recommendations regarding which measures of predictive ability should be reported [5,10,11]. Measures such as the c-statistic and the measures of reclassification do not capture the consequences of implementing a prediction model. Hence, we do not recommend their use in evaluating which new predictor may prove to be clinically useful in a particular population as these measures assess model fit rather than clinical utility. We recommend that a measure such as net benefit is calculated and the results adjusted to allow for the difference in the mean risk between the study population and the population in which the new predictor will be implemented. If benefits and costs are to be measured over a specific time period a measure such as the EFLY should be used which accounts for the different costs and benefits that would be accrued over time in populations with different mean risks.

## Abbreviations

NRI: Net reclassification improvement; NRI(>0): Continuous net reclassification improvement; ROC: Receiver operator characteristic; AUC: Area under the curve; EFLY: Event free life years; SBP: Systolic blood pressure; CVD: Cardiovascular disease.

## Competing interests

The authors declare that they have no competing interests.

## Authors’ contributions

KM designed the study, carried out the analysis, interpreted the results and drafted the manuscript. The other authors, PM, LI and PB, all contributed to the interpretation of results, reviewed the manuscript and revised it for important intellectual content. All authors read and approved the final manuscript.

## Pre-publication history

The pre-publication history for this paper can be accessed here:

http://www.biomedcentral.com/1471-2288/14/86/prepub

## Supplementary Material

Additional file 1 **Table S1.** Hazard ratios for the addition of systolic blood pressure to models predicting CVD for men and women in Framingham study. **Figure S1.** NRI(10%, 20%) for selected combinations of baseline model and new predictor. **Figure S2.** Difference in c-statistics for selected combinations of baseline model and new predictor. Click here for file
