# Supplementary material for: An assessment of the relationship between clinical utility and predictive ability measures and the impact of mean risk in the population
Source: BMC Med Res Methodol. 2014 Jul 3;14:86. doi: 10.1186/1471-2288-14-86 (PMC4105158; doi:10.1186/1471-2288-14-86)
Supplement: Additional file 1 — Table S1. Hazard ratios for the addition of systolic blood pressure to models predicting CVD for men and women in Framingham study. Figure S1. NRI(10%, 20%) for selected combinations of baseline model and new predictor. Figure S2. Difference in c-statistics for selected combinations of baseline model and new predictor. [file 1471-2288-14-86-S1.docx]

**Additional file 1: Table S1: Hazard ratios for the addition of systolic blood pressure to models predicting CVD for men and women in Framingham study**

|  | Men |  |  |  | Women |  |  |  |
| --- | --- | --- | --- | --- | --- | --- | --- | --- |
|  | Base Model | | Base Model + systolic blood pressure | | Base Model | | Base Model + systolic blood pressure | |
|  | Hazard ratio (95% CI) |  | Hazard ratio (95% CI) |  | Hazard ratio (95% CI) |  | Hazard ratio (95% CI) |  |
| Age (per 1 standard deviation increase) | 2.04 (1.88, 2.21) |  | 1.89 (1.74, 2.06) |  | 1.96 (1.75, 2.19) |  | 1.64 (1.46, 1.85) |  |
| Total cholesterol (per 1 standard deviation increase) | 1.25 (1.16, 1.34) |  | 1.23 (1.14, 1.32) |  | 1.30 (1.18, 1.42) |  | 1.25 (1.14, 1.37) |  |
| HDL (per 1 standard deviation increase) | 0.79 (0.73, 0.86) |  | 0.78 (0.72, 0.85) |  | 0.82 (0.74, 0.91) |  | 0.83 (0.75, 0.91) |  |
| Hypertensive medication | 1.72 (1.41, 2.08) |  | 1.45 (1.18, 1.77) |  | 1.76 (1.41, 2.19) |  | 1.31 (1.04, 1.65) |  |
| Current smoker | 1.91 (1.64, 2.22) |  | 1.93 (1.66, 2.24) |  | 1.71 (1.40, 2.07) |  | 1.72 (1.42, 2.09) |  |
| Diabetes | 1.89 (1.53, 2.34) |  | 1.77 (1.42, 2.19) |  | 2.14 (1.60, 2.86) |  | 2.07 (1.55, 2.77) |  |
| Systolic blood pressure (per 1 standard deviation increase) |  |  | 1.26 (1.18, 1.35) |  |  |  | 1.48 (1.34, 1.62) |  |

**Additional file 1: Figure S1**: NRI(10%, 20%) for selected combinations of baseline model and new predictor


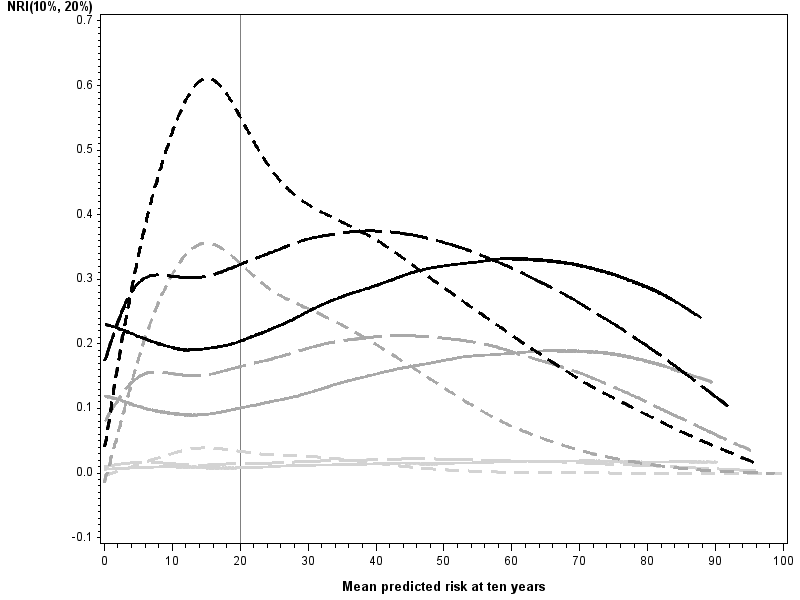


Legend: Strong baseline + strong new predictor

Medium baseline + strong new predictor

Weak baseline + strong new predictor

Strong baseline + medium new predictor

Medium baseline + medium new predictor

Weak baseline + medium new predictor

Strong baseline + weak new predictor

Medium baseline + weak new predictor

Weak baseline + weak new predictor

**Additional file 1: Figure S2**: Difference in c-statistics for selected combinations of baseline model and new predictor


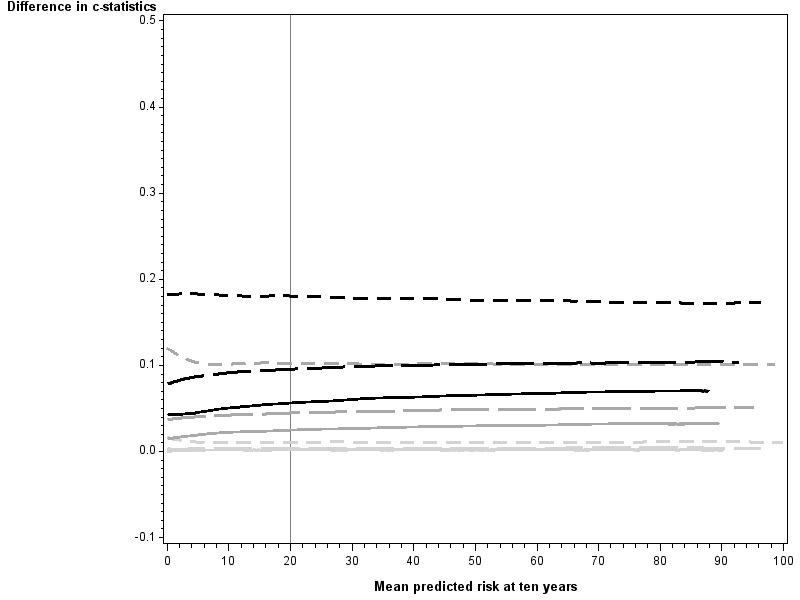


Legend: Strong baseline + strong new predictor

Medium baseline + strong new predictor

Weak baseline + strong new predictor

Strong baseline + medium new predictor

Medium baseline + medium new predictor

Weak baseline + medium new predictor

Strong baseline + weak new predictor

Medium baseline + weak new predictor

Weak baseline + weak new predictor
